# Pinpointing the *PRDM9-PRDM7* Gene Duplication Event During Primate Divergence

**DOI:** 10.3389/fgene.2021.593725

**Published:** 2021-02-24

**Authors:** Sacha Heerschop, Zahra Fagrouch, Ernst J. Verschoor, Hans Zischler

**Affiliations:** ^1^Division of Anthropology, Institute of Organismic and Molecular Evolution, Faculty of Biology, Johannes Gutenberg University Mainz, Mainz, Germany; ^2^Department of Virology, Biomedical Primate Research Centre, Rijswijk, Netherlands

**Keywords:** *PRDM7*, *PRDM9*, gene duplication, paralogization, primate evolution

## Abstract

Studies on the function of *PRDM9* in model systems and its evolution during vertebrate divergence shed light on the basic molecular mechanisms of hybrid sterility and its evolutionary consequences. However, information regarding *PRDM9*-homolog, *PRDM7*, whose origin is placed in the primate evolutionary tree, as well as information about the fast-evolving DNA-binding zinc finger array of strepsirrhine PRDM9 are scarce. Thus, we aimed to narrow down the date of the duplication event leading to the emergence of *PRDM7* during primate evolution by comparing the phylogenetic tree reconstructions of representative primate samples of PRDM orthologs and paralogs. To confirm our *PRDM7* paralogization pattern, database-deposited sequences were used to test the presence/absence patterns expected from the paralogization timing. In addition, we extended the existing phylogenetic tree of haplorrhine *PRDM9* zinc fingers with their strepsirrhine counterparts. The inclusion of strepsirrhine zinc fingers completes the *PRDM9* primate phylogeny. Moreover, the updated phylogeny of *PRDM9* zinc fingers showed distinct clusters of strepsirrhine, tarsier, and anthropoid degenerated zinc fingers. Here, we show that *PRDM7* emerged on the branch leading to the most recent common ancestor of catarrhines; therefore, its origin is more recent than previously expected. A more detailed character evolutionary study suggests that *PRDM7* may have evolved differently in Cercopithecoidea as compared to Hominoidea: it lacks the first four exons in Old World monkeys orthologs and exon 10 in Papionini orthologs. Dating the origin of *PRDM7* is essential for further studies investigating why Hominoidea representatives need another putative histone methyltransferase in the testis.

## Introduction

*PRDM9* is a putative speciation gene consisting of the KRAB, SSXRD, and SET domains and a zinc finger array (Hayashi et al., 2005; Birtle and Ponting, 2006). Its expression is restricted to male and female germlines during meiosis I (Hayashi et al., 2005). *PRDM9* has been studied in several haplorrhine primate species (Oliver et al., 2009; Auton et al., 2012; Groeneveld et al., 2012; Schwartz et al., 2014; Heerschop et al., 2016; Stevison et al., 2016). The zinc finger array of this gene has undergone rapid evolution with regard to the number of tandem repeats as well as its DNA-binding amino acids, and has been associated with positive selection (Oliver et al., 2009; Thomas et al., 2009; Myers et al., 2010). Phylogenies of *PRDM9* show that the 5′-most zinc fingers of all studied primates are distinct from other canonical C2H2 zinc finger sequences (Schwartz et al., 2014; Heerschop et al., 2016).

During primate evolution, a duplication event led to the emergence of *PRDM9*′s homolog, *PRDM7*, presumably on the branch of Haplorrhini or Anthropoidea (Fumasoni et al., 2007; Vervoort et al., 2016). Due to the recency of this event, the two genes are highly similar in some aspects. While the PR/SET domains are having a 97% identical sequence, structural differences are present downstream of the PR domain (Blazer et al., 2016). *PRDM7* shows an internal duplication of 89 bases in human exon 10 according to the transcript *PRDM7*-202 (Ensembl, transcript ID: ENST00000449207.7). This duplication is not based on repetitive elements, and leads to a frameshift and a new splicing site, which gives rise to some isoforms expressing four zinc fingers, while others do not express any zinc fingers (Fumasoni et al., 2007). While *PRDM7* is mainly expressed in testis, expression patterns were observed in other tissues such as melanocytes (Hayashi et al., 2005; Fumasoni et al., 2007). Compared to *PRDM9*, *PRDM7* exhibits a reduced catalytic function on H3K4me3 and has no catalytic function on H3K36me3. Three missense mutations in the PR domain of *PRDM7*, particularly the change from tyrosine to serine at position 357, might have led to these functional differences (Blazer et al., 2016). Fumasoni et al. (2007) suggested that *PRDM9* is the ancestor gene of PRDM7 because its genomic surroundings and its gene and protein structures are more conserved in comparison to its paralog PRDM7.

The main goal of this paper is to narrow down the origin of *PRDM7* with phylogenetic reconstructions and additional amplified sequences to support the findings. A second aim is to include strepsirrhine *PRDM9* zinc fingers in an existing phylogeny to reveal if they form a cluster and, therefore, present a similar pattern of concerted evolution as haplorrhine zinc fingers.

## Methods

### Phylogeny of *PRDM7* and *PRDM9*

Two strategies were applied to determine the origin of *PRDM7* during primate divergence. The underlying sequences of the two phylogenetic reconstructions were obtained via BLASTN with normal search sensitivity, Ensembl version 102. The first one contains exon 7 to exon 9 including introns. Exon 7 and exon 9 of human *PRDM9* were blasted within all primate species on Ensembl, and the sequences from the start of exon seven to the end of exon nine were downloaded. With BLASTN and “distant homologies” as search sensitivity, the homolog sequences of *Bos taurus* and *Mus musculus* were obtained as outgroups. The second phylogenetic reconstruction is based on the human *PRDM9* 3’ UTR which was also blasted within all primate species, extended to 1500 bases downstream and downloaded. In both cases, only BLAST results with 70% of the length and e-values below 0.0001 were accepted. The loci of the analyzed sequences and the genome assemblies can be found in Supplementary Data Sheets 1 and 2. If possible *PRDM7* and *PRDM9* were assigned with the help of syntenic regions on Ensembl. In cercopithecoid species, similar patterns in the BLAST results of *PRDM7* exons (see section “Ensembl BLAST”) were used to distinguish the genes. The phylogenetic reconstruction was performed on ngphylogeny.fr (Dereeper et al., 2008; Lemoine et al., 2019) with the following workflow: MAFFT was used for the alignment (Katoh and Standley, 2013), Gblocks for the curation (Castresana, 2000; Talavera and Castresana, 2007), PhyML with 1000 bootstraps as support for the tree inference (Guindon et al., 2010; Lemoine et al., 2018) with default settings (substitution model: GTR + I + G) besides the bootstrap support and Newick for tree rendering (Junier and Zdobnov, 2010). Adjustments were made with the Interactive Tree of Life (iTOL) v4 (Letunic and Bork, 2019): nodes with bootstrap values below 500 were collapsed. *B. taurus* was set as outgroup in the phylogeny based on exons and introns.

### PCRs of *PRDM7* and *PRDM9*

For additional support, we used PCR to amplify the last exon of *PRDM7* and *PRDM9* (primer: 5′-ACATCTACCCTGACCA AAAAC-3′, 5′-CGGATTTGTTTAATCAGTTATTTC-3′) of *Macaca mulatta* (Tm: 52°C), *Hylobates lar* (Tm: 50°C), *Pongo abelii* (Tm: 52°C), *Gorilla gorilla gorilla* (Tm: 54°C), *Pan troglodytes* (Tm: 52°C), and *Homo sapiens* (Tm: 52°C). The differences in length were observed on an agarose gel with the Thermo Scientific GeneRuler 100 bp Plus DNA Ladder: *PRDM7* gives a short amplicon and *PRDM9* can give one or two longer amplicons due to length polymorphisms in the zinc finger array. We Sanger sequenced all three products of *M. mulatta* and used a BLAST routine to check whether the amplicons overlap with *PRDM7* or *PRDM9*. Furthermore, we used PCR to amplify a sequence within exon 10 of PRDM7, containing a *PRDM7-*specific internal duplication for following species: *Daubentonia madagascariensis*, *Cheirogaleus medius*, *Carlito syrichta*, *Callithrix jacchus*, *H. lar*, *P. abelii*, *G. g. gorilla*, *P. troglodytes*, and *H. sapiens* (primer: 5′-AACTGTGCCCGGGATGAT-3′, 5′-ACTTGCTGCCCCACTTGAT-3′, Tm: 51°C). Samples from six non-related individuals per species (*G. g. gorilla* and *P. troglodytes*) were amplified. Human data on *PRDM7*’s internal duplication and *PRDM9*’s corresponding sequences were retrieved from the 1000 Genomes Project (Auton et al., 2015) by using an in-house shell-script. All exons were concatenated, and the unique sequences were selected with the help of in-house Python scripts (see Supplementary Data Sheet 1).

### Ensembl BLAST

All human *PRDM7*_202 exons were blasted against the genomes of *M. mulatta*, *Macaca fascicularis*, *Macaca nemestrina*, *Theropithecus gelada*, *Papio anubis*, *Cercocebus atys*, *Mandrillus leucophaeus*, *Chlorocebus sabaeus*, *Rhinopithecus roxellana*, *Rhinopithecus bieti*, *Piliocolobus tephrosceles*, *Nomascus leucogenys*, *P. abelii*, *G. g. gorilla*, and *P. troglodytes* on Ensembl version 102, with default settings (Yates et al., 2020). For Cercopithecoidea, another BLAST with human *PRDM9*_201 exon 10 was performed. To evaluate the consequences of missing exons, human *PRDM7* exons 5–11 were analyzed with ScanProsite (Sigrist et al., 2002; Castro et al., 2006) as well as human *PRDM7* lacking exon 10 (based on the Papionini sequence).

The Segmental Duplication Database (Bailey et al., 2001, 2002; She et al., 2004a,b) was searched to see if *PRDM7* and *PRDM9* are listed. To check if *PRDM9* and *PRDM7* differ in their predicted loss of function variants, both genes were scanned on Genome Aggregation Database (gnomAD), v2.1.1 (Karczewski et al., 2020).

### Phylogeny of *PRDM9* Zinc Fingers

The reconstruction of a *PRDM9* zinc finger phylogeny is based on sequence data from Heerschop et al. (2016) and data from Schwartz et al. (2014). It encompasses zinc fingers of nine strepsirrhine species, including *Microcebus murinus*, *C. medius*, *Eulemur coronatus*, *Prolemur simus*, *Varecia variegata*, *Propithecus coquereli*, *D. madagascariensis*, *Nycticebus coucang*, and *Otolemur garnettii* and one degenerated zinc finger of the mouse and cow as outgroups. Sequences of *C. medius*, *E. coronatus*, *V. variegata*, *D. madagascariensis*, and *N. coucang* were obtained by sequencing PCR products amplified with the following primers: 5′-GCCCAGAACAGGCCAGA-3′ and 5′-TTCTGGTCTCTTTACACTCTTGG-3′ (Tm: 50°C, *D. madagascariensis* and *E. coronatus*); 5′-GGGATCAGAATCAGGAGCAG-3′ and 5′-CTCTAGTCAT GAAAGTAGAGGATTTG-3′ (Tm: 54°C, *C. medius*, *V. variegata*, and *N. coucang*). The shorter alleles of *D. madagascariensis* and *E. coronatus* were separated by gel electrophoresis and extracted using the QIAquick^®^ Gel Extraction Kit of Qiagen. One allele of *V. variegata* was isolated by ligation into a pGEM T-vector and transfected into TOP10 electrocompetent cells. *PRDM9* exon 11 zinc finger array sequences of *C. medius*, *D. madagascariensis*, *E. coronatus*, and *V. variegata* have been deposited in GenBank under the accession numbers MT862444–MT862447. The sequence of *N. coucang* can be found in Supplementary Data Sheet 1. Data on *M. murinus* (*PRDM9*, ENSMICG00000037613), *P. simus* (*PRDM7*, ENSPSMG00000016627), *P. coquereli* (*PRDM7*, ENSPCOG00000013284), and *O. garnettii* (GL873654:27557–28889) were extracted by looking for human PRDM9 exon 11 orthologs using BLAST on the Ensembl database, version 102. Although listed as *PRDM7*, we assumed that the sequences of *P. coquereli* and *O. garnettii* represent *PRDM9*. A zinc finger alignment was prepared manually, and DNA binding codons were removed. The phylogenetic reconstruction was performed on ngphylogeny.fr (Dereeper et al., 2008; Lemoine et al., 2019) with following workflow: MAFFT was used for the alignment (Katoh and Standley, 2013), BMGE for the curation (Criscuolo and Gribaldo, 2010), PhyML with SH-like support for the tree inference (Guindon et al., 2010; Lemoine et al., 2018) with default settings (substitution model: GTR + I + G) beside the support and Newick for tree rendering (Junier and Zdobnov, 2010). Adjustments were made with iTOL v4 (Letunic and Bork, 2019): nodes with SH-like values below 0.5 were collapsed. *B. taurus* was set as outgroup. A pruned phylogenetic tree of primate zinc fingers was constructed by merging the zinc fingers of Anthropoidea and Tarsiers, and editing the Newick file manually.

## Results

### The Origin of *PRDM7*

**FIGURE 1:**
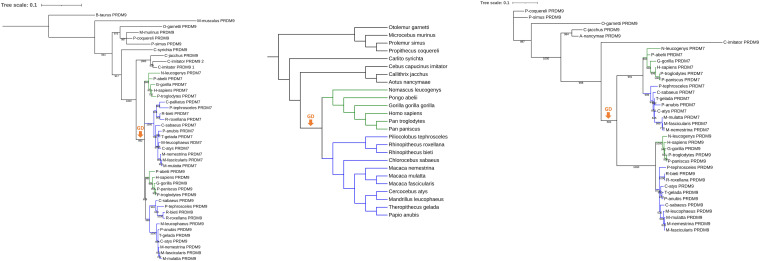
The origin of *PRDM7*. The phylogenetic reconstructions on primate *PRDM7* and *PRDM9* sequences based on exon seven to nine (left) and the 1500 bp downstream of the stop codon (right) show the gene duplication event, marked with an orange arrow. In both cases, *PRDM9* sequences of Catarrhini form a cluster and *PRDM7* sequences of Cercopithecoidea (blue) and Hominoidea (green) are the sister taxa (left) or taxon (right). That suggests that *PRDM7* originated on the branch of catarrhine before the split of Cercopithecoidea and Hominoidea. *PRDM9* of *Bos taurus* was chosen as the outgroup in the phylogeny based on exons 7–9. The bootstrap values of nodes are displayed. The scale bar indicates the mean number of nucleotide substitutions per site. The phylogeny in the middle displays a species tree that was created based on Geissmann (2003) and Perelman et al. (2011).

Phylogenetic reconstructions of *PRDM7* and *PRDM9* orthologs were performed to determine when *PRDM7* arose. PCRs of the last exon should confirm the results. The basis of the presented phylogenetic reconstructions is represented by sequencing data obtained from the Ensembl genome browser, version 102. Both phylogenetic reconstructions yield similar topological results (Figure 1). *PRDM7* of diverse catarrhine species forms a monophylum in the 3′ phylogeny. In the pylogeny including exons and introns, *PRDM7* of Cercopithecoidea and Hominoidea cluster separately as sister taxa to *PRDM9* of Catarrhini. *PRDM7* branches off in the phylogenies following the Platyrrhini–Catarrhini split. The phylogenetic pattern representing *PRDM9* in Hominoidea, Cercopithecoidea, Platyrrhini, and Strepsirrhini resembles the topology of the widely accepted species tree. The BLAST for exons 7 and 9 shows two results in *Cebus capucinus imitator* and they form sister taxa in the phylogeny. In the phylogeny based on the 3′ UTR, the second *PRDM9* was not integrated because of unassembled regions.

**FIGURE 2:**
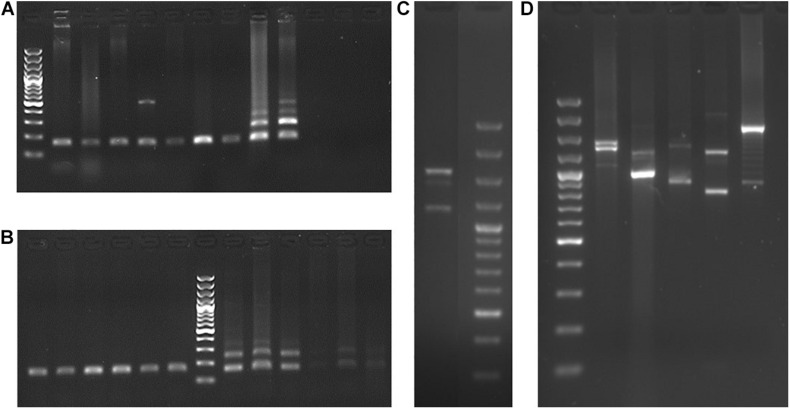
Characterization of *PRDM7* and *PRDM9* in primates. PCR products of *PRDM7* and *PRDM9* for **(A)** exon 10 of *D. madagascariensis*, *C. medius*, *C. syrichta*, *C. jacchus*, *H. lar*, *P. abelii*, *G. g. gorilla*, *P. troglodytes*, and *H. sapiens* show that the internal duplication in *PRDM7* is exclusive to chimp and human. **(B)** Exon 10 of several, mostly unrelated gorillas (left side of the ladder) and chimpanzees (right side of the ladder) reveals that there is no incomplete lineage sorting regarding the internal duplication in *PRDM7*. **(C)** Exon 11 of *M. mulatta*. **(D)** Exon 11 of *H. lar*, *P. abelii*, *G. g. gorilla*, *P. troglodytes*, and *H. sapiens*. The last two gels show both, *PRDM7* and *PRDM9* amplicons in these anthropoid species. The Thermo Scientific GeneRuler 100 bp Plus DNA Ladder was used.

The PCR amplification of exon 11 of *M. mulatta*, *H. lar*, *P. abelii*, *G. g. gorilla*, *P. troglodytes*, and *H. sapiens* exhibited two or three products representing *PRDM7* and *PRDM9* (Figures 2C,D). The presence of two *PRDM9* alleles with length polymorphisms explains the amplification of a third product. Sequencing of these *M. mulatta* products and subsequent BLASTs indicate that the shortest sequence represents the *PRDM7* and the two longer sequences represent the *PRDM9* alleles.

The regions chr5: 23504220–23528686 and ch16: 90123418–90147718 are listed in the Segmental Duplication Database and match with the loci of *PRDM9* (chr5: 23507724–23528706) and *PRDM7* (chr16: 90122974–90142338) of the human genome assembly GRCh37 on which the Segmental Duplication Database is based.

According to gnomAD, the expected and observed predicted losses of function variants are similar for both genes, with values of 1.01 for PRDM9 and 1.02 for PRDM7.

### Internal Duplication of *PRDM7* Is Exclusively Found in Human and Chimp

PRDM7 displays an internal duplication in human. Our aim is to check whether this is a distinct marker to distinguish PRDM7 from PRDM9 in primates. The PCR amplification of the region encompassing *PRDM7*’s internal duplication showed that only the *P. troglodytes* and *H. sapiens* samples displayed products of two distinct sizes, thus differing from a variety of strepsirrhine and haplorrhine species of primates (Figure 2A). To further support this observation, we amplified this region in five non-related gorillas and four non-related chimpanzees to exclude the possibility of incomplete lineage sorting of ancestral polymorphisms. The gorilla samples showed one product, while for the chimpanzee samples, we observed at least two products (Figure 2B). Human data obtained from the 1000 Genomes Project confirmed our PCR results; while 246 non-identical *PRDM7* sequences showed internal duplication in exon 10, the 74 non-identical *PRDM9* sequences showed the shorter version of exon 10.

### Absent *PRDM7* Exons in Catharrhini

A BLAST search of human *PRDM7* (PRDM7_202) exons, examining the genome assemblies of 11 Cercopithecoidea and four Hominoidea representatives from the Ensembl database, was performed to screen for differences in these taxa. It generated two hits with the expected length, indicating the high sequence similarity between *PRDM7* and *PRDM9*. No hit was obtained for *PRDM7* exons 1–4 in the 11 Cercopithecoidea and Gibbon genomes. Furthermore, exons 1–6 of chimpanzee *PRDM7* were not listed in the BLAST results. The sequences upstream of exons 5 and 7 contain unassembled sites in the cases of *C. sabaeus*, *R. roxellana*, *T. gelada*, *N. leucogenys*, and *P. troglodytes*. Nevertheless, the BLAST analysis of *P. abelii* and *G. g. gorilla* yielded all exons. In addition, macaques and *P. anubis* lack exon 10, while the sequence between exons 9 and 11 of both species revealed no unassembled sections. The lengths of these sequences were 1718 bp in *P. anubis*, 3502 bp in *M. mulatta*, and 2917 bp in *H. sapiens*. The BLAST results for Cercopithecoidea showed two *PRDM7* exon 10 hits (Table 1), which are due to the insert of 89 bp in exon 10 (found in chimpanzees and humans). *N. leucogenys* lacks exon 7 and 10 in its *PRDM9* locus. The BLAST query sequence with human *PRDM9* exon 10 resulted in a better fit with one hit. Furthermore, two hits for exon 11 were obtained for Cercopithecoidea. This might indicate their origin from rudimentary zinc finger repeats.

**TABLE 1:** PRDM7 exons in five Old World monkeys.

**Exon**	**Length (bp)**	**Mm, chr20**	**Pa, chr20**	**Tg, chr20**	**Rr, KN294467**	**Cs, chr5**
1	98	x	x	x	X	x
2	154	x	x	x	X	x
3	124	x	x	x	X	x
4	108	x	x	x	X	x
5	50	77127057–77127106	72029987–72030036	47728119–47728168	376720–376769	75395647–75395696
6	157	77123503–77123657	72025315–72025469	47724578–47724732	373502–373656	75392430–75392584
7	102	77122294–77122393	72024106–72024205	47723369–47723468	372293–372392	75391222–75391321
8	272	77121819–77122089	72023634–72023898	47722893–47723163	371825–372083	75390746–75391016
9	68	77121350–77121417	72023165–72023232	47722427–47722491	371356–371423	75390277–75390344
10	283	x	x	47720856–47721005	370226–370377	75389147–75389298
				47720814–47720942	370184–370316	75389105–75389237
11	755	77117248–77117847	72020847–72021446	47717833–47718432	367766–368431	75386576–75387244
		77117176–77117370	72020775–72020969	47717761–47717955	367761–367879	75386406–75386685

*BLAST results of human *PRDM7* exons against Mm: *Macaca mulatta*, Pa: *Papio anubis*, Tg: *Theropithecus gelada*, Rr: *Rhinopithecus roxellana*, and Cs: *Chlorocebus sabaeus*. All species show no results for exons 1–4. Exon 10 is missing in *M. mulatta* and *P. anubis*. For each exon, the locus in the genome of each species is given. “x” indicates no hits. A more detailed overview can be found in Supplementary Data Sheet 3.*

A ScanProsite analysis of manually truncated human *PRDM7* amino acid sequences revealed that exons 2–4 encode the KRAB domain and exon 10 partly encodes the SET domain.

### Missing Cluster of Strepsirrhine Zinc Fingers

**FIGURE 3:**
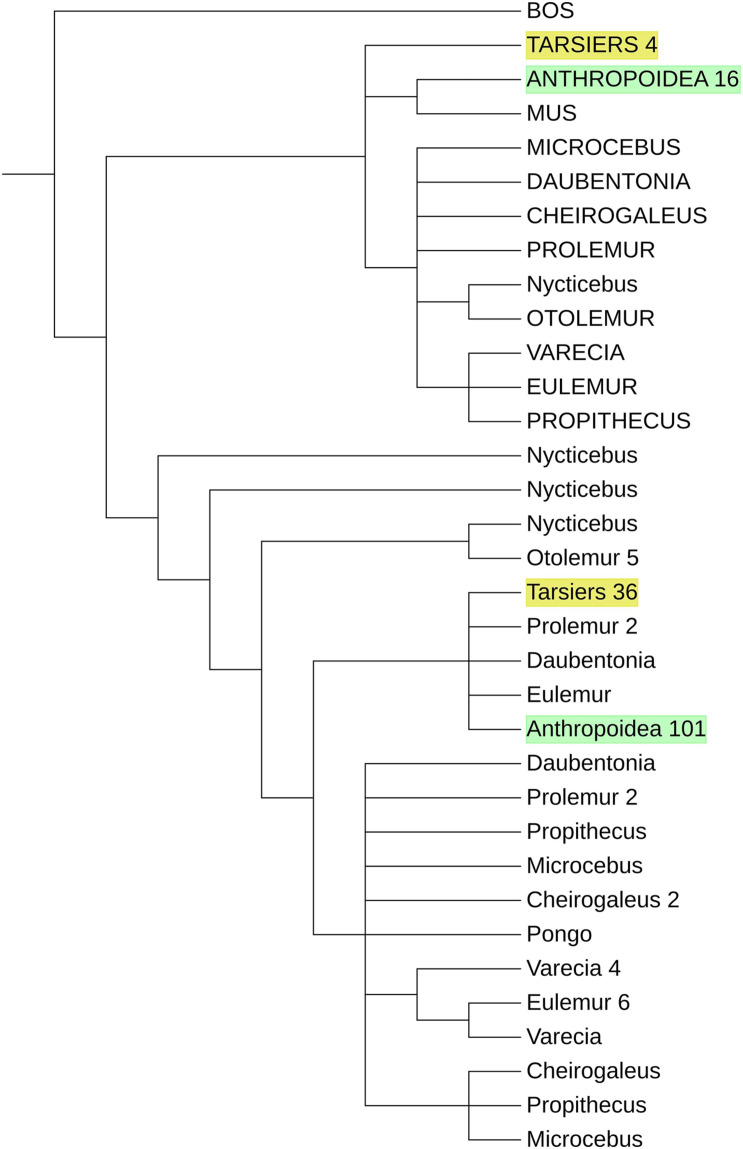
Schematic phylogeny of primate *PRDM9* zinc fingers. The degenerated zing fingers of Strepsirrhini, Tarsier, and Anthropoids form clusters. The C2H2 zinc fingers of the strepsirrhine primates are the sister taxa of the other primates, but they do not show a distinct clustering. Sequences of Anthropoidea and Tarsiers are merged with the number of zinc fingers indicated at the end of the identifier. Degenerated zinc fingers are displayed in capital letters, and the zinc fingers of Anthropoidea and Tarsiers are marked in green and yellow, respectively. See Supplementary Data Sheet 1 for a more detailed version.

We integrated strepsirrhine *PRDM9* zinc finger sequences into a previously published data set to see if they form a distinct cluster like Tarsiers and Anthropoids do. The phylogeny of *PRDM9* zinc fingers (Figure 3) showed three clusters of 5′ end degenerated zinc fingers belonging to the strepsirrhine, tarsier, and anthropoid taxa with a C2H2 zinc finger of *N. coucang* as sister taxa of *O. garnettii* degenerated zinc finger. In contrast, the classical zinc fingers of Strepsirrhini do not form a cluster. C2H2 zinc fingers of *O. garnettii* and *N. coucang* form outgroups to all other C2H2 zinc fingers. With the exceptions of one *D. madagascariensis*, one *E. coronatus*, and two of *P. simus* orthologs, Lemuriformes zinc fingers branch off from the lineage leading to haplorrhine C2H2 zinc fingers. Tarsier classical zinc fingers form a monophylum in the haplorrhine cluster.

## Discussion

By including strepsirrhine data to the phylogeny of *PRDM9* zinc fingers previously published by Heerschop et al. (2016), we herein show that strepsirrhines contrast the haplorrhine primates by not forming a cluster as Tarsiers or Anthropoids do. Therefore, the realization of a concerted evolution of the zinc fingers as proposed by Schwartz et al. (2014) for the anthropoid primates does not apply for the *PRDM9* zinc fingers of Strepsirrhini, especially regarding C2H2 zinc fingers of the two Lorisiformes representatives, *O. garnettii* and *N. coucang*. They branch off earlier in the phylogeny than those of the other strepsirrhine primates. This may indicate different patterns and regimes governing *PRDM9* zinc finger evolution in the two branches (Lemurs and Loris) representing the deepest split of Strepsirrhini.

The date of *PRDM7’*s origin is narrowed down to the lineage leading to extant Catarrhini, after the platyrrhines split off. The well-supported topology obtained from the phylogenetic reconstructions is corroborated by presence/absence analyses. Accordingly, no signature of a paralogization event leading to *PRDM7* was observed in platyrrhine species following a BLAST query of the genomes of several representatives of these species. The corresponding period dates back to an estimated 32–43 million years ago, and since then several segmental duplications arose in catarrhine primates as indicated by the analysis of the human genome (Lander et al., 2001; Samonte and Eichler, 2002; Perelman et al., 2011). These segmental duplications are not evenly distributed throughout chromosomes, thus being predominantly located in the pericentromeric and subtelomeric regions (Bailey et al., 2001; Lander et al., 2001). Due to *PRDM7’*s position at the end of chromosome 16q and its putative emergence during a period of segmental duplication, it is reasonable to speculate that this gene probably originated from the aforementioned rearrangements. These assumptions support the previously established hypothesis that portrays *PRDM9* as its ancestral gene. Fumasoni et al. (2007) concluded that *PRDM9* is the predecessor of *PRDM7* based on the conserved genomic flanks and the high similarity of rodent co-orthologs.

The internal duplication of *PRDM7* is an exclusive characteristic of chimpanzees and humans and is therefore not suitable for distinguishing *PRDM7* from *PRDM9* in other species. Nevertheless, the effects of this frameshifting rearrangement on the function of *PRDM7* with and without the duplication remain to be determined. The BLAST searches of human *PRDM7* exons querying catarrhine genomes revealed that cercopithecine primates and *N. leucogenys* might lack the first four exons, while *P. troglodytes* might lack six exons. It remains unclear whether these exons are missing or the inability to detect them is due to poorly assembled segments. Furthermore, *PRDM7* exon 10 is missing in macaques and *P. anubis*. *T. gelada*, *M. leucophaeus*, and *C. atys* are more closely related to *P. anubis* than they all are to macaques. However, the BLAST results of exon 10 show a match in the sister taxa of *P. anubis*. Here is yet to be determined if information is lacking or if there were two separate events leading to the loss of exon 10 in macaques and *P. anubis*. Exons 2–4 are coding for the KRAB domain, while exon 10 codes partially for the SET domain. Therefore, *PRDM7* is putatively experiencing a loss of functional constraints on the cercopithecine branch.

The most common result of a gene duplication is the pseudogenization of one copy (Ohno, 1972). While *PRDM7* might be a pseudogene in Old World monkeys, its putative H3K4 methyltransferase activity, shown in a catalytic assay and in cells with human *PRDM7* cDNA (Blazer et al., 2016), and the homologous structure in gorilla and orangutan could indicate that there is a sub- or neofunctionalization of this young PRDM member in Hominoidea family. However, due to similar branch lengths during both PRDM7- and PRDM9-divergence, both positive selection and loss of functional constraints apparently did not play a major role after paralogization and early evolution of the duplicate. Similarly, character evolution as revealed by the relative proportion of functional substitutions obtained by querying the gnomAD database for both human *PRDM7* and *PRDM9* does not point toward signatures of selection on the population level, too. *PRDM9* has an essential function in meiosis by determining hotspot loci and is a putative speciation gene (Mihola et al., 2009; Baudat et al., 2010; Myers et al., 2010). However, some mammals do not have functional *PRDM9* copies, like dogs (Oliver et al., 2009; Muñoz-Fuentes et al., 2011). In human, loss of function did not result in sterility as shown in a case of a mother (Narasimhan et al., 2016).

The available data on primate genomes are limited. Most primate genome assemblies are on a level of scaffolds, for example, the *C. imitator* assembly. There is a possibility that with new and improved data and coverage of primate diversity, the outcome may differ from ours. However, the results shown in this article are strong evidence that *PRDM7* originated on the branch of Catarrhini. Therefore, genes annotated as *PRDM7* in non-catarrhine species are probably *PRDM9*, exemplified in *P. coquereli* on Ensembl. We also suggest that PRDM7 in ruminants (Ahlawat et al., 2016) is a different PRDM member and possibly resulted from another duplication event.

Based on the data and interpretations herein, species with a putative functional *PRDM7* can be reasonably well defined and further studies can focus to elucidate the function of this recent PRDM member.

## Data Availability Statement

The datasets presented in this study can be found in online repositories. The names of the repository/repositories and accession number(s) can be found in the article/Supplementary Material.

## Author Contributions

SH performed the experiments and the analysis and wrote the manuscript. SH and HZ planned the study and discussed the results. ZF extracted the DNA of the non-related chimpanzees and gorillas. ZF and EV provided samples. EV and HZ revised the manuscript. All authors read and approved the final manuscript.

## Conflict of Interest

The authors declare that the research was conducted in the absence of any commercial or financial relationships that could be construed as a potential conflict of interest.

## Supplementary Material

Supplementary Data Sheet 1 Partial sequences of PRDM9 and PRDM7 exon 11 of Macaca mulatta as well as the zinc finger array of PRDM9 of Nycicebus coucang. A detailed phylogeny on the PRDM9 zinc fingers. A table on primate species, the corresponding genome assemblies and loci of PRDM7 and PRDM9. In-house scripts to work with data of the 1000 genomes project.

Supplementary Data Sheet 2 Blasted Loci for the phylogenies on PRDM7 and PRDM9.

Supplementary Data Sheet 3 Ensembl BLAST results of human PRDM7 exons.
