# Supplementary material for: Pinpointing the PRDM9-PRDM7 Gene Duplication Event During Primate Divergence
Source: Front Genet. 2021 Feb 24;12:593725. doi: 10.3389/fgene.2021.593725 (PMC7943923; doi:10.3389/fgene.2021.593725)
Supplement: Supplementary Data Sheet 1 — Partial sequences of PRDM9 and PRDM7 exon 11 of Macaca mulatta as well as the zinc finger array of PRDM9 of Nycicebus coucang. A detailed phylogeny on the PRDM9 zinc fingers. A table on primate species, the corresponding genome assemblies and loci of PRDM7 and PRDM9. In-house scripts to work with data of the 1000 genomes project. [file Data_Sheet_1.docx]

**Supplements**

1. **Supplementary Data**

> Macaca_mulatta_Exon11_1600bp

CCATGTCCCTCATGCTGTCTGGCCTTTTCAAGTCAGAAATTTCTCAGTCACCACGTGGAACGCAATCACTCCACTCAGAACTTCCGAGGACCATCTGCAAGAAGACTCCTCCAACCAGAGAATCTCTGCTCAGGGGATCAGAATCAGGAGCAGCAATATTCTGATCCACGCAGCTGTAATGACAAAACCAAAGGTCAAGAGATCAAAGAAAGGTCCAAACTCTTGAATAAAAGGACATGGCAGAAGGAGATTTCAAGGGCCTTTTCTAGCCCACCCAAAGGACAAATGGGGAGCTCTAGAGTGGGAGAAAGAATGATGAAAGAAGAGTACAGAACAGGCCAGAAAGTGAATCCAGAGAACACAGGCAAATTATTTGTGGGAGTAGGAATCTCAAGAATTGCAAAAGTCAAGTATGGAGAGTGTGGGCAAGGTTTCAGTGATAAATCAGATGTTATTATACACCAGAGGACACACACAGGGGAGAAGCCCTACCTCTGCAGGGAGTGTGGGCGGGGCTTTAGCCGGAAGTCAAACCTCCTCTGTCACCAGAGGACACACACAGGGGAGAAGCCCTACCTCTGCAGGGAGTGTGGGCGGGGCTTTAGAGATAATTCAAGCCTCCGCTATCACCAGAGGACACACACAGGGGAGAAGCCCTACCTGGCCAGGGAGTGTGGGCGGGGCTTTAGCGATAATTCAGGCCTCCGCTATCACCAGAGGACACACACAGGGGAGAAGCCCT

> Macaca_mulatta_Exon11_1500bp

ATGTCCCTCATGCTGTCTGGCCTTTTCAAGTCAGAAATTTCTCAGTCAACATGTGGAACGCAATCACTCCACTCAGAACTTCCCAGGGCCATCTGCAAGAAGACTCTTCCAACCAGAGAATCTCTGCTCAGGGGATCAGAATCAGGAGCAGCAATATTCTGATCCACGCAGCTGTAATGACAAAACGAAAGGTCAAGAGATCAAAGAAAGGTCCAAACTCTTGAATAAAAGGACATGGCCGAAGGAGATTTCAAGGGCCTTTTCTAGCCCACCCAAAGGACAAATGGGGAGCTCTAGAGTGGGAGAAAGAATGATGGAAGAAGAGTACAGAACAGGCCAGAAAGTGAATCCAGAGAACACAGGCAAATTATTTGTGGGAGTAGGAATCTCAAGAATTGCAAAAGTCAAGTATGGAGAGTGTGGGCAAGGTTTCAGTGATAAATCAGATGTTATTATACACCAGAGGACACACACAGGGGAGAAGCCCTACCTCTGCAGGGAGTGTGGGCGGGGCTTTAGCCAGAAGTCAAGCCTCCGCCGTCACCAGAGGACACACACAGGGGAGAAGCCCTACCTCTGCAGGGAGTGTGGGCGGGGCTTTAGAGATAATTCAAGCCTCCGCTATCACCAGAGGACACACACAGGGGAGAAGCCCTACCTCTGCAGGGAGTGTGGGCGGGGCTTTAGCAATAATTCAGG

>Macaca_mulatta_Exon11_1200bp

ATTCTCGTTCTGTCCCCCAGGCTGGAGTGCAGTGGCCGGATCTCAGCTCACTGCAAGCTCCACCTCTCGGGTTCACGCCATTCTCCTGCCTCAGCCTCCCGAGTAGCTGGGACTACAGGCGCCCGCCACCGCTCCCGGCTAATTTTTGTATTTTTTTAGTAGAGACGGGGTTTCACGTGTTAGCCAGGATGGTCTCGATCTCCTGAACTCGTGATCCGCCCGTCTCGGCCTCCCAAAGTGCTGGGATTACAGGCTTGAGCCACCGCGCCCGGCCAAAACTTCCTCTTTCAGAACCAAAGCCAGAGATCTATCCATGCCCCTCATGCTGTCTGGCCTTTTCAAGTCAGAAATTTCTGTCAACATGTGGAACGCAATCACTCCTCTCAGAACTTCCCAGGACCATCTGCAAGAAAACTCCTCCAATCAGAGAATCCCTGCCCAGGGGATCAGAATCAGGAGCAGCAATATTCTGATCCATCCAGCTGTAATGACAAAACCAAAGGTCAAGAGATCAAAGAAAGGTCCAAACTCTTGAATAAAAGGACATGGCAGAGGGAGATTCTAAGGGCTTTTACTAGCCCACCCAAAGGACAAATGGGGAGCTCTAGAGTGGGAGAAAGAATGATGGAAGAAGAGTTCAGAACAGGCCAGAAAGCGAAT

CCAGGGAACACGGGCAAATTATTTGTGGGGGTAGAAATCTCAAGAATTGCAAAAGTCAAGTATGGAGAGTGTGGGCAAGGTTTCAGTGGTAAGTCAGATGTTATTACACACCAAAGGACACACACAGAGGGGAAGCCCTATGTCTGCAGGGGGTGTGGGCGGCGCTTTAGCCAGAAGTCAAGCCTCCTCAGACACCAGAGGACACACACAGGGGAGAAGCCCTAGTCTGCAAGAAGAATGAGTAAGTCTTTAGTAATAAAACCTTATCTCAATAGCCACAAGAAGACAAATGTGGTCACCACACATTTGCACACCCCAGCTCTGAGGTGGCTTCAGCGGAAATCTGCTGACCCCTTGC

>Nycticebus coucang_Znf exon

GATTTCAAGGATATTTTCCTGCCCACCTAAGGGACAAATGGGGAGCTCTAGGGAGGGTGAAAGAATGATAGAGGAAGAGCCCAGACGAGrCCAGAAAGTGGGTCCAGGGGATACAGGAGAATTATGTGTGACAGTAGGGATCTCAAGAATTATAAAAGTCAAGAATGGAAAGTGTGGGCAAAGTTTCAGTGATAAGTAAAACCTCCTCACACACAGATGACACACACAGGGGAGAAGCCCTACATC**TGCAGGAAGTGTGGACAACCATTTAGCCATAAGTCAACCCTCCTCACACACCAGACGACACACATAGGGGAGAAGCCGTACATTTGCAGGGAGTGTGGGCAAGGATTTAGCCAAAAGCCACACCTCATCACACACCAGAGGACGCACACAGAGGAGAAGCCCTATGTCTGCAGGGACTGTGGGCAGAGCTTCACTGATAAATCATCCCTCCTCAGACACCAGAGGACACACACAGGGGAAAAGCCCTATATCTGCAGGGACTGTGGGCGAAGATTTAGCCAAAAGCCACACCTCATCACACATCAGAGGACACACAGAGAAGCCCTACATCTGCAG**GGACTGTGGGCGAGGATTTAGCCGTAAGTCAAACCTCCCTACACACCAGAGGACACACACAAATACCTATGCTTTCAAGAAGGGTTAGTAAGTCATTAGTAATAAAAAyATCTCAACAGCCACAAGAGGGCAAATGTGGCCATAACACACCTCCACACCTTAGCTCTGAGAGGGCTTTACAGGAAGTCTCCTGACCCCTTAGATTCCCCAAAAGTGTAAACAGCAGAAATAACTGATTAATCAAATCCTCTACTTC

Note: The zinc fingers are marked in bold type. There are stop codons 5’ to the zinc finger array, possibly evaded by alternative splicing.

1. **Supplementary Figures**


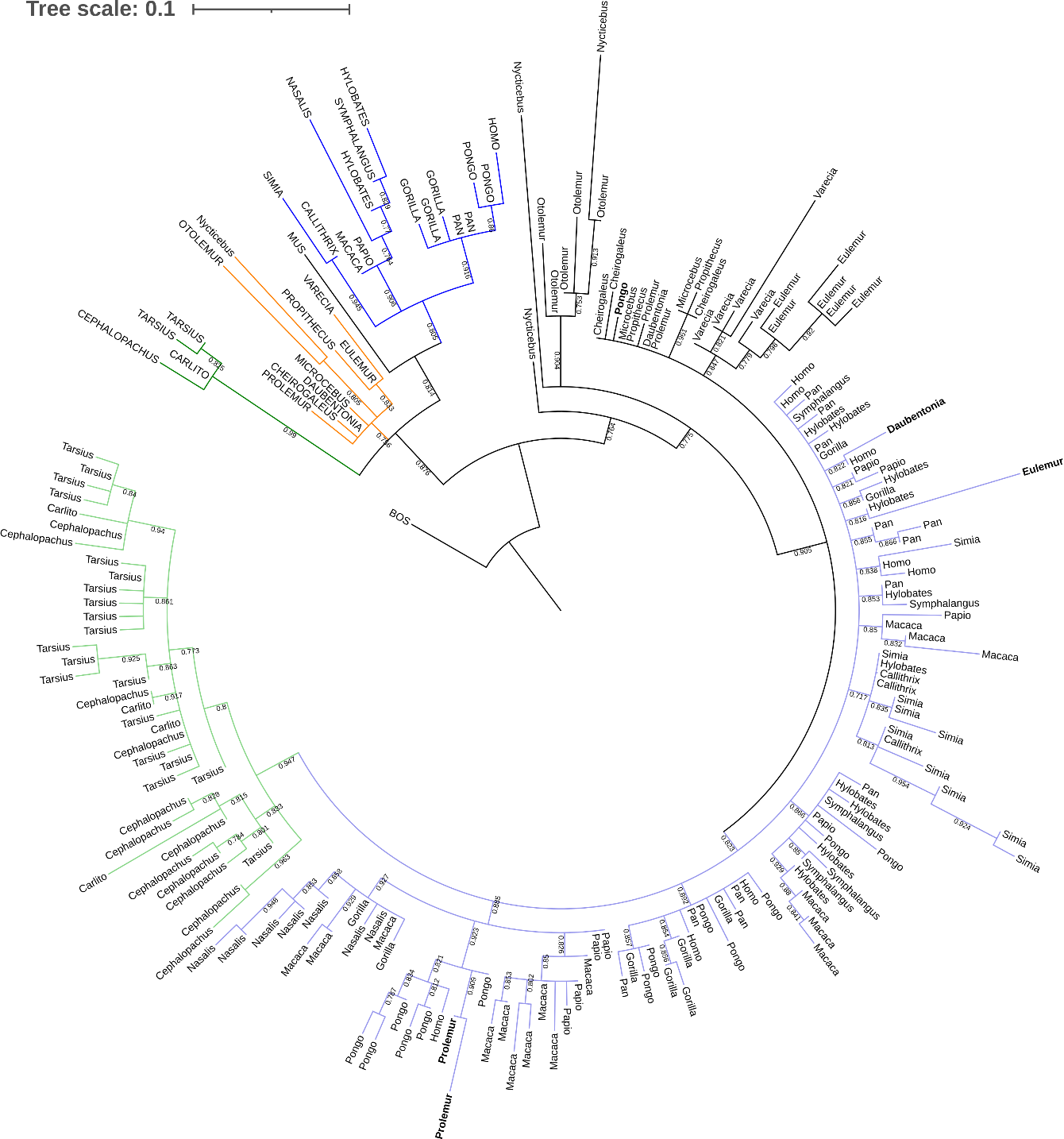


Supplementary figure 1: Phylogeny of primate PRDM9 zinc finger, based on previous phylogenies of Schwartz et al. (2014) and Heerschop et al. (2016). Within in the degenerated zinc finger (all capital letters), there are three clusters: tarsiers (green), strepsirrhini (orange) and anthropoids (blue). The classical C2H2 zinc fingers of tarsiers (light green) and anthropoids (light blue) also cluster with those of strepsirrhine forming sister taxa. Strepsirrhine zinc fingers in the anthropoid cluster and Pongo among strepsirrhine zinc fingers are marked bold type. The degenerated zinc finger of Bos taurus was chosen as outgroup, the one of Mus musculus forms a sister clade to the anthropoid degenerated zinc finger cluster. SH-like values are presented at the nodes. The scale bar indicates the mean number of nucleotide substitutions per site. Displayed species: Homo – *Homo sapiens*; Pan – *Pan troglodytes*, *Pan paniscus*; Gorilla – *Gorilla gorilla gorilla*, *Gorilla gorilla beringei*; Pongo – *Pongo abelii*, *Pongo pygmaeus*; Hyobates – *Hylobates pileatus*, *Hylobates gabriellae*, *Hylobates leucogenys*; Symphalangus – *Symphalangus syndactilus*; Nasalis – *Nasalis larvatus*; Papio – *Papio cynocephalus*; Macaca – *Macaca mulatta*, *Macaca nemestrina*; Simia – *Simia lagotricha*; Callithrix – *Callithrix jacchus*; Carlito – *Carlito syrichta*; Cephalopachus – *Cephalopachus bancanus*; Tarsius – *Tarsius dentatus*, *Tarsius lariang*, *Tarsius fuscus*, *Tarsius sp.*; Microcebus – *Microcebus murinus*; Cheirogaleus – *Cheirogaleus medius*; Eulemur – *Eulemur coronatus*; Prolemur – *Prolemur simus*; Varecia – *Varecia variegata*; Propithecus – *Propithecus coquereli*; Daubentonia – *Daubentonia madagascariensis*; Nycticebus – *Nycticebus coucang*; Otolemur – *Otolemur garnettii*.

1. **Supplementary Tables**

Supplementary table 1: Ensembl genome assemblies, species and genes used for phylogenetic reconstructions and associated locus.

| **Name** | **Genome assembly** | **Gene** | **Locus [chromosome or scaffold]** |
| --- | --- | --- | --- |
| Aotus nancymaae | Anan_2.0 (GCA_000952055.2) | >A.nancymae_PRDM9 | >scaffold:Anan_2.0:KZ199641.1 |
| Cercocebus atys | Caty_1.0 (GCA_000955945.1) | >C.atys_PRDM7 | >scaffold:Caty_1.0:KQ009501.1 |
|  |  | >C.atys_PRDM9 | >scaffold:Caty_1.0:KQ010425.1 |
| Cebus capucinus imitator | Cebus_imitator-1.0 (GCA_001604975.1) | >C.imitator_PRDM9 | >scaffold:Cebus_imitator-1.0:KV390448.1 |
| Callithrix jacchus | ASM275486v1 (GCA_002754865.1) | >C.jacchus_PRDM9 | >primary_assembly:ASM275486v1:NTIC01034390.1 |
| Chlorocebus sabaeus | ChlSab1.1 (GCA_000409795.2) | >C.sabaeus_PRDM7 | >chromosome:ChlSab1.1:5 |
|  |  | >C.sabaeus_PRDM9 | >chromosome:ChlSab1.1:4 |
| Carlito syrichta | Tarsius_syrichta-2.0.1 (GCA_000164805.2) | >C.syrichta_PRDM9 | >scaffold:Tarsius_syrichta-2.0.1: |
| Gorilla gorilla gorilla | gorGor4 (GCA_000151905.3) | >G.gorilla_PRDM7 | >chromosome:gorGor4:16 |
|  |  | >G.gorilla_PRDM9 | >chromosome:gorGor4:17 |
| Homo sapiens | GRCh38.p13 (GCA_000001405.28) | >H.sapiens_PRDM7 | >chromosome:GRCh38:16 |
|  |  | >H.sapiens_PRDM9 | >chromosome:GRCh38:5 |
| Macaca fascicularis | Macaca_fascicularis_5.0 (GCA_000364345.1) | >M.fascicularis_PRDM7 | >chromosome:Macaca_fascicularis_5.0:20 |
|  |  | >M.fascicularis_PRDM9 | >chromosome:Macaca_fascicularis_5.0:6 |
| Mandrillus leucophaeus | Mleu.le_1.0 (GCA_000951045.1) | >M.leucophaeus_PRDM7 | >scaffold:Mleu.le_1.0:KN972985.1 |
|  |  | >M.leucophaeus_PRDM9 | >scaffold:Mleu.le_1.0:KN978117.1 |
| Macaca mulatta | Mmul_10 (GCA_003339765.3) | >M.mulatta_PRDM7 | >primary_assembly:Mmul_10:20 |
|  |  | >M.mulatta_PRDM9 | >primary_assembly:Mmul_10:6 |
| Microcebus murinus | Mmur_3.0 (GCA_000165445.3) | >M.murinus_PRDM9 | >chromosome:Mmur_3.0:20 |
| Macaca nemestrina | Mnem_1.0 (GCA_000956065.1) | >M.nemestrina_PRDM7 | >scaffold:Mnem_1.0:KQ005136.1 |
|  |  | >M.nemestrina_PRDM9 | >scaffold:Mnem_1.0:KQ008744.1 |
| Nomascus leucogenys | Nleu_3.0 (GCA_000146795.3) | >N.leucogenys_PRDM7 | >chromosome:Nleu_3.0:2 |
|  |  | >N.leucogenys_PRDM9 | >chromosome:Nleu_3.0:6 |
| Otolemur garnettii | OtoGar3 (GCA_000181295.3) | >O.garnettii_PRDM9 | >scaffold:OtoGar3:GL873654.1 |
| Pongo abelii | PPYG2 | >P.abelii_PRDM7 | >chromosome:PPYG2:16 |
|  |  | >P.abelii_PRDM9 | >chromosome:PPYG2:5 |
| Papio anubis | Panu_3.0 (GCA_000264685.2) | >P.anubis_PRDM7 | >chromosome:Panu_3.0:20 |
|  |  | >P.anubis_PRDM9 | >chromosome:Panu_3.0:6 |
| Propithecus coquereli | Pcoq_1.0 (GCA_000956105.1) | >P.coquereli_PRDM9 | >scaffold:Pcoq_1.0:KQ025192.1 |
| Pan paniscus | panpan1.1 (GCA_000258655.2) | >P.paniscus_PRDM7 | >chromosome:panpan1.1:16 |
|  |  | >P.paniscus_PRDM9 | >chromosome:panpan1.1:5 |
| Prolemur simus | Prosim_1.0 (GCA_003258685.1) | >P.simus_PRDM9 | >primary_assembly:Prosim_1.0:MPIZ01002088.1 |
| Piliocolobus tephrosceles | ASM277652v2 (GCA_002776525.2) | >P.tephrosceles_PRDM7 | >primary_assembly:ASM277652v2:PDMG02000277.1 |
|  |  | >P.tephrosceles_PRDM9 | >primary_assembly:ASM277652v2:PDMG02000177.1 |
| Pan troglodytes | Pan_tro_3.0 (GCA_000001515.5) | >P.troglodytes_PRDM7 | >chromosome:Pan_tro_3.0:16 |
|  |  | >P.troglodytes_PRDM9 | >chromosome:Pan_tro_3.0:5 |
| Rhinopithecus bieti | ASM169854v1 (GCA_001698545.1) | >R.bieti_PRDM7 | >scaffold:ASM169854v1:MCGX01000445.1 |
|  |  | >R.bieti_PRDM9 | >scaffold:ASM169854v1:MCGX01012500.1 |
| Rhinopithecus roxellana | Rrox_v1 (GCA_000769185.1) | >R.roxellana_PRDM7 | >scaffold:Rrox_v1:KN294467.1 |
|  |  | >R.roxellana_PRDM9 | >scaffold:Rrox_v1:KN298576.1 |
| Theropithecus gelada | Tgel_1.0 (GCA_003255815.1) | >T.gelada_PRDM7 | >primary_assembly:Tgel_1.0:20 |
|  |  | >T.gelada_PRDM9 | >primary_assembly:Tgel_1.0:6 |

1. **Supplementary Scripts**

**Script 1:** It samples all sequences of a defined region from the 1000 Genomes Project. Here *PRDM9* is the example.

#!/bin/sh

for sample in `bcftools view -h PRDM9.1000g.vcf.gz | grep "^#CHROM" | cut -f10-`; do

bcftools view -c1 -Oz -s $sample -o 1000g.$sample.vcf.gz PRDM9.1000g.vcf.gz

tabix -p vcf 1000g.$sample.vcf.gz

samtools faidx human_g1k_v37.fasta.gz 5:23505626-23530804 \

| bcftools consensus 1000g.$sample.vcf.gz -o PRDM9.1000g.$sample.fa

done

**Script 2**: It extracts the given exons and concatenates them. In this example it is *PRDM9*

# division necessary for right calaculation, so everything below 1 is calculated exactly and not as 1

from __future__ import division

import os

# exons to find

exon1 = "CTCTGAGAGAACGCCCGGCCAGGGTGAACGCCGCGGCAGGAGAGCACGGGAGATTGTGAAGAGCATGGGGAGCCTTTGTCGTGCAGCGTGAAACCCTT"

exon2 = "CACCTTCTCCTTCCACAGGAGCCTTTGGCCTAGGAGCTGGGAGACTCAGGGCCCTTCTCACACTCAGAATTGGAGCAGGGCCTTCTAGACAGTCCCAGCACCATGAGCCCTGAAAAGTCCCAAGAGGAGAGCCCAGAAGAAGACACAGAGAGAACAGAGCGGAAGCCCATG"

exon3 = "GTCAAAGATGCCTTCAAAGACATTTCCATATACTTCACCAAGGAAGAATGGGCAGAGATGGGAGACTGGGAGAAAACTCGCTATAGGAATGTGAAAAGGAACTATAATGCACTGATTACTATAG"

exon4 = "GTCTCAGAGCCACTCGACCAGCTTTCATGTGTCACCGAAGGCAGGCCATCAAACTCCAGGTGGATGACACAGAAGATTCTGATGAAGAATGGACCCCTAGGCAGCAAG"

exon5 = "TCAAACCTCCTTGGATGGCCTTAAGAGTGGAACAGCGTAAACACCAGAAG"

exon6 = "GGAATGCCCAAGGCGTCATTCAGTAATGAATCTAGTTTGAAAGAATTGTCAAGAACAGCAAATTTACTGAATGCAAGTGGCTCAGAGCAGGCTCAGAAACCAGTGTCCCCTTCTGGAGAAGCAAGTACCTCTGGACAGCACTCAAGACTAAAACTGG"

exon7 = "AACTCAGGAAGAAGGAGACTGAAAGAAAGATGTATAGCCTGCGAGAAAGAAAGGGTCATGCATACAAAGAGGTCAGCGAGCCGCAGGATGATGATTACCTCT"

exon8 = "ATTGTGAGATGTGTCAGAACTTCTTCATTGACAGCTGTGCTGCCCATGGGCCCCCTACATTTGTAAAGGACAGTGCAGTGGACAAGGGGCACCCCAACCGTTCAGCCCTCAGTCTGCCCCCAGGGCTGAGAATTGGGCCATCAGGCATCCCTCAGGCTGGGCTTGGAGTATGGAATGAGGCATCTGATCTGCCGCTGGGTCTGCACTTTGGCCCTTATGAGGGCCGAATTACAGAAGACGAAGAGGCAGCCAACAATGGATACTCCTGGCTG"

exon9 = "ATCACCAAGGGGAGAAACTGCTATGAGTATGTGGATGGAAAAGATAAATCCTGGGCCAACTGGATGAG"

exon10 = "GTATGTGAACTGTGCCCGGGATGATGAAGAGCAGAACCTGGTGGCCTTCCAGTACCACAGGCAGATCTTCTATAGAACCTGCCGAGTCATTAGGCCAGGCTGTGAACTGCTGGTCTGGTATGGGGATGAATACGGCCAGGAACTGGGCATCAAGTGGGGCAGCAAGTGGAAGAAAGAGCTCATGGCAGGGAGAG"

exon11 = "AACCAAAGCCAGAGATCCATCCATGTCCCTCATGCTGTCTGGCCTTTTCAAGTCAGAAATTTCTCAGTCAACATGTAGAACGCAATCACTCCTCTCAGAACTTCCCAGGACCATCTGCAAGAAAACTCCTCCAACCAGAGAATCCCTGCCCAGGGGATCAGAATCAGGAGCAGCAATATCCAGATCCACACAGCCGTAATGACAAAACCAAAGGTCAAGAGATCAAAGAAAGGTCCAAACTCTTGAATAAAAGGACATGGCAGAGGGAGATTTCAAGGGCCTTTTCTAGCCCACCCAAAGGACAAATGGGGAGCTGTAGAGTGGGAAAAAGAATAATGGAAGAAGAGTCCAGAACAGGCCAGAAAGTGAATCCAGGGAACACAGGCAAATTATTTGTGGGGGTAGGAATCTCAAGAATTGCAAAAGTCAAGTATGGAGAGTGTGGACAAGGTTTCAGTGTTAAATCAGATGTTATTACACACCAAAGGACACATACAGGGGAGAAGCTCTACGT"

# exonlist to iterate through

exonlist = [exon1, exon2, exon3, exon4, exon5, exon6, exon7, exon8, exon9, exon10, exon11]

# in case of SNPs: hamming distance, compares two sequences, each difference is counted

def hammingdistance(seq1, seq2):

count = 0

for i, j in zip(seq1, seq2):

if i != j:

count += 1

return count

# opens direction, import os

folder_list = os.listdir("C:/Users/path")

# cut header, fa-files: header is not seperated by new line; in this case the header contains no letters

def cutheader(fafile):

sequ = ""

for i in fafile:

if i.isalpha():

sequ += i

return sequ

# findexons searches in each sequence for all exons in exonlist

def findexons():

for files in folder_list: # iterates through all files in folderlist/directory

filea = open(os.path.join("C:/Users/path/", files), "r").read() # open files

seq = cutheader(filea) # function cutheader is used to get the sole sequence

cdna = "" # exons are put here

for exon in exonlist: # searches each exon of exonlist in seq

for i in range(0, len(seq) - len(exon) + 1): #

if seq[i: (i + len(exon))] == exon: # if exon is identical with part of seq

cdna += seq[i: (i + len(exon))] # part of seq is concatenated to cdna

break # if exon is found, the search is stopped because there is probably no duplicate. saves time

elif (hammingdistance(seq[i: (i + len(exon))], exon) / len(exon)) < 0.1: # if exon differs slightly with part of seq, 1 exchange in 10 bp -> 0.1

cdna += seq[i: (i + len(exon))] # part of seq is concatenated to cdna

break # if exon is found, the search is stopped because there is probably no duplicate. saves time

output = open(str(files) + "_cdna.fas", "w") # new file for cdna should be called like old file + _cdna

output.write(">" + str(files) + "\n" + cdna + "\n") # file contains header [>filename], nextline: cdna

output.close()

findexons()

**Script 3:** It removes identical sequences and counts all non-identical sequences.

# find all identical sequences and list them with their seq. count haplotypes.

import os

folder_list = os.listdir("C:/Users/path")

d = {} # dictionary for all sequences and their header

for files in folder_list:

filea = open(os.path.join("C:/Users/path/", files), "r")

file_lines = filea.readlines()

header = file_lines[0].strip()

seq = file_lines[1].strip()

d[header] = seq # dictionary, header as key snd seq as value

haplolist = [] # list with non-identical sequences

count = 0

for key, value in d.iteritems():

if value not in haplolist: # is sequence not in list:

haplolist.append(value) # puts non-identical sequences in list

count += 1 # count and number as identifier for identical sequences / identification number

if count < 10:

number = "00" + str(count) # to assure that all numbers have three digits, easier to sort

elif 9 < count < 100:

number = "0" + str(count) # to assure that all numbers have three digits, easier to sort

else:

number = str(count) # to assure that all numbers have three digits, easier to sort

output = open("identicalsP9.txt", "a") # text file with

output.write(str(number) + "_" + str(key) + "haplo" + " " + str(value) + "\n")

output2 = open("haploseqP9.fas", "a") # fasta file with all haplotypes

output2.write(str(key) + "haplo" + str(number) + "\n" + str(value) + "\n")

output2.close()

elif value in haplolist: # in case of sequence being already in haplolist

number2 = haplolist.index(value) + 1 # identification number

if number2 < 10:

number3 = "00" + str(number2) # identification number with three digits

elif 9 < number2 < 100:

number3 = "0" + str(number2) # identification number with three digits

else:

number3 = str(number2) # identification number with three digits

output = open("identicalsP9.txt", "a") # appends all header names with identification number when their sequence is already in haplolist

output.write(str(number3) + str(key) + "\n")

output.close()

sort_file = open("C:/Users/path/identicalsP9.txt", "r").readlines()

output3 = open("identicalsP9sorted.txt", "w") # file with non-identical sequences and corresponding headers sorted by identification number

output3.write("\n".join(sorted(set(sort_file))))

output3.close()
